# *Drosophila melanogaster*: A platform for anticancer drug discovery and personalized therapies

**DOI:** 10.3389/fgene.2022.949241

**Published:** 2022-08-08

**Authors:** Chamoné Munnik, Malungi P. Xaba, Sibusiso T. Malindisa, Bonnie L. Russell, Selisha A. Sooklal

**Affiliations:** ^1^ Department of Life and Consumer Sciences, University of South Africa, Pretoria, South Africa; ^2^ Buboo (Pty) Ltd, The Innovation Hub, Pretoria, South Africa

**Keywords:** *Drosophila melanogaster*, cancer models, high-throughput screening, drug discovery, personalized therapy

## Abstract

Cancer is a complex disease whereby multiple genetic aberrations, epigenetic modifications, metabolic reprogramming, and the microenvironment contribute to the development of a tumor. In the traditional anticancer drug discovery pipeline, drug candidates are usually screened *in vitro* using two-dimensional or three-dimensional cell culture. However, these methods fail to accurately mimic the human disease state. This has led to the poor success rate of anticancer drugs in the preclinical stages since many drugs are abandoned due to inefficacy or toxicity when transitioned to whole-organism models. The common fruit fly, *Drosophila melanogaster*, has emerged as a beneficial system for modeling human cancers. Decades of fundamental research have shown the evolutionary conservation of key genes and signaling pathways between flies and humans. Moreover, *Drosophila* has a lower genetic redundancy in comparison to mammals. These factors, in addition to the advancement of genetic toolkits for manipulating gene expression, allow for the generation of complex *Drosophila* genotypes and phenotypes. Numerous studies have successfully created *Drosophila* models for colorectal, lung, thyroid, and brain cancers. These models were utilized in the high-throughput screening of FDA-approved drugs which led to the identification of several compounds capable of reducing proliferation and rescuing phenotypes. More noteworthy, *Drosophila* has also unlocked the potential for personalized therapies. *Drosophila* ‘avatars’ presenting the same mutations as a patient are used to screen multiple therapeutic agents targeting multiple pathways to find the most appropriate combination of drugs. The outcomes of these studies have translated to significant responses in patients with adenoid cystic carcinoma and metastatic colorectal cancers. Despite not being widely utilized, the concept of *in vivo* screening of drugs in *Drosophila* is making significant contributions to the current drug discovery pipeline. In this review, we discuss the application of *Drosophila* as a platform in anticancer drug discovery; with special focus on the cancer models that have been generated, drug libraries that have been screened and the status of personalized therapies. In addition, we elaborate on the biological and technical limitations of this system.

## 1 Introduction

Cancer is one of the leading causes of death worldwide, claiming approximately 10 million lives in 2020. The disease represents a major global burden with 19.3 million new cases reported in 2020 (Sung et al., 2021). With these numbers expected to increase, it demonstrates the urgent need for intervention in detection and treatment strategies. Cancer is a complex disease whereby multiple genetic aberrations, epigenetic modifications, metabolic reprogramming, and the microenvironment contribute to the development of a tumor. Despite advances in understanding the molecular drivers of cancer, our ability to translate anticancer research into clinical success has been poor. The progression of new anticancer drugs through the drug discovery pipeline has one of the highest failure rates in comparison to other diseases. In effect, less than 5% of anticancer drugs that are developed ultimately reach the market (Moreno and Pearson, 2013; Liu et al., 2017). Several factors contribute to this high failure rate, most notably is the complexity of the disease and limitations in preclinical screening tools which fail to simulate these complexities.

**FIGURE 1:**
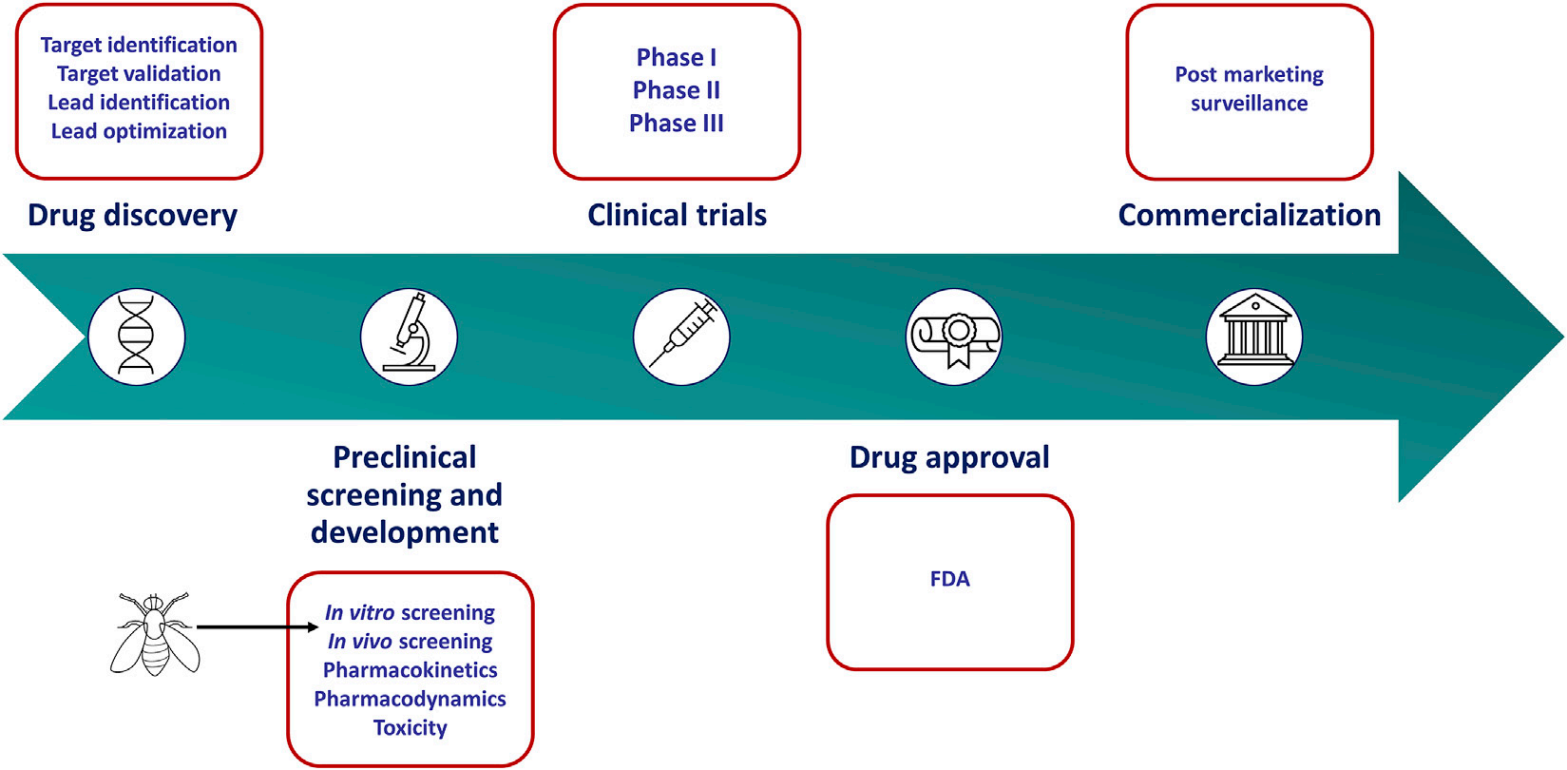
Drug discovery and development pipeline. *In vitro* culture screens have become a standard tool used in the preclinical stages to identify anticancer agents. However, these models fail to mimic key characteristics of human tumors. *Drosophila melanogaster* can recapitulate these characteristics more accurately and may serve as a more appropriate screening tool in the preclinical stages of the drug discovery pipeline.

In the traditional anticancer drug discovery pipeline, *in vitro* screening has become a standard tool used in the preclinical stages to identify candidate drugs with clinical potential (Figure 1). Small-molecule candidates are typically screened for activity using two-dimensional or three-dimensional cell culture. Two-dimensional (2-D) cell culture offers high-throughput screening with extensive panels of cell lines representing various cancer types. However, these cultures which are often found in monolayer, do not adequately represent key characteristics of human tumors such as the three-dimensional (3-D) architecture of tumors and the host-tumor environment. Furthermore, prolonged culturing of cancer cell lines leads to genetic drift. Consequently, cell lines may exhibit substantial genetic, epigenetic, and phenotypic variations induced by an artificial environment and therefore may not reflect the original tumor (Hoelder et al., 2012; Verjans et al., 2018). Three-dimensional cell culture, in the form of spheroids and organoids, was thus designed to resemble *in vivo* tumors more closely. These cell cultures offer the opportunity for a more in-depth study of characteristics such as cellular contact, the extracellular matrix, drug penetration, nutrient distribution, and aspects of drug resistance (Richardson et al., 2015; Horvath et al., 2016). Several factors, including the cost and reproducibility, limit the application of three-dimensional cell culture. For instance, different culture techniques often give rise to spheroids with varying sizes and shapes. This has been found to influence drug efficacy and toxicity (Zanoni et al., 2016). In addition, it is challenging to utilize this culture technique in a high-throughput manner (Verjans et al., 2018). Both 2-D and 3-D cell culture screening methods fail to accurately mimic the host-tumor microenvironment and recapitulate the complex mechanisms found *in vivo*. Consequently, many positive hits identified using these types of *in vitro* screens are abandoned in the preclinical stages due to inefficacy or toxicity when transitioned to whole-organism models (Horvath et al., 2016; Adams et al., 2021). This highlights the shortcomings of *in vitro* cell culture in the evaluation of anticancer drugs and emphasizes the need for more physiologically relevant screening tools that capture the properties of the cancer cell as well as the host-tumor microenvironment.

The common fruit fly, *Drosophila melanogaster*, has emerged as a beneficial platform for investigating cancer. *Drosophila* has typically been utilized as a pathway discovery platform, enabling the successful identification of key components in cancer-related pathways such as WNT, HIPPO, JAK/STAT, RAS, NOTCH, HEDGEHOG, BMP and TGF-β (Rudrapatna et al., 2012; Cagan et al., 2019). However, apart from being a powerful pathway identification tool, the organism has also attracted attention as an anticancer drug screening tool. Conservation of key genes, a low genetic redundancy, development of genetic manipulation toolkits and the rapid life cycle of *Drosophila* have provided a selective advantage for modeling human tumors as well as whole-organism anticancer drug screening (Cagan et al., 2019). It has been estimated that approximately 75% of genes affiliated with a disease in humans, have functional homologs in *Drosophila* (Wangler et al., 2017). In a cancer-specific context, these include key genes involved in the cell cycle, differentiation, cell migration, cell polarity, cell adhesion and apoptosis. It is significant to point out that these genes appear at a lower frequency in *Drosophila* due to the lower genetic redundancy observed in comparison to mammals. This feature has proven advantageous for drug discovery since fewer genes need to be manipulated in order to set up a sensitized condition for drug screening (Su, 2019). These factors, in addition to the advancement of genetic toolkits for manipulating gene expression allow for the generation of complex *Drosophila* genotypes and phenotypes (Cagan et al., 2019; Su, 2019). Therefore, *Drosophila* can depict the cancer state more accurately than traditional *in vitro* cell culture systems, and has the added advantage of the host-tumor environment. *Drosophila* also offers many practical advantages. The organism has a rapid life cycle (∼ 10 days) with the ability to produce large numbers of offspring and is relatively cheap and easy to maintain. Moreover, both larvae and mature adults are small enough to fit into 96-well microtiter plates. This makes the organism suitable for high-throughput screening of large drug libraries (Richardson et al., 2015).

The translation of anticancer drug activity, determined in the preclinical screening stages, into efficacy in the clinic remains a major hurdle. The factors associated with the failure of such anticancer drugs have been reviewed elsewhere (Jardim et al., 2017). The burden of wasted money and resources applied into development of a drug that fails to show therapeutic relevance provides a strong motivation to re-consider our preclinical screening practices (Hoelder et al., 2012). *Drosophila* has the potential to recapitulate some hallmarks of cancer unerringly and the ability to be utilized in a high-throughput screening manner. Moreover, screening using *Drosophila* provides information on drug bioavailability, toxicity to the organism and host-tumor interactions. As such, *Drosophila* represents a far more valuable screening tool in the preclinical stages of anticancer drug discovery. Given the anatomical differences between *Drosophila* and humans, this system cannot be used to study all cancer types. Therefore, *Drosophila* may not replace whole-organism mammalian models but can serve as a more appropriate screening tool than *in vitro* cultures for particular cancer types (Figure 1). The application of this organism in the anticancer drug discovery pipeline is still in its infancy, and greater awareness and utilization should be encouraged. Herein, we discuss the application of *Drosophila* as a platform in anticancer drug discovery; with special focus on the cancer models that have been generated, drug libraries that have been screened and the status of personalized therapies. In addition, we elaborate on the biological and technical limitations of this system, placing it in context as an amenable tool for drug discovery.

## 2 *Drosophila* models in cancer types

**FIGURE 2:**
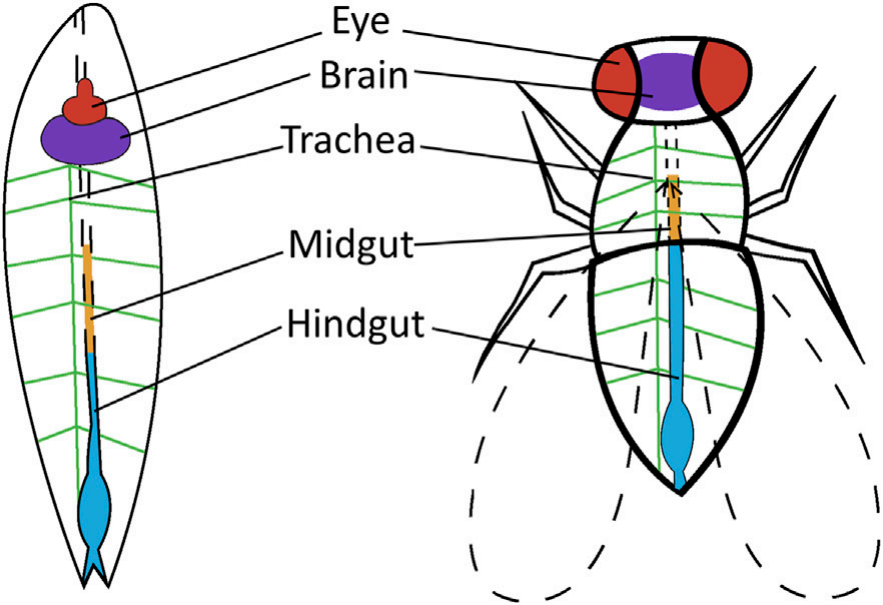
*Drosophila* tissue or organs used for cancer research. Tissue or organs are shown in the *Drosophila* larvae and *Drosophila* adult that are utilized in human cancer research. Thyroid cancer is targeted to the eye of adult *Drosophila* (red). Brain cancer is targeted to the brain of adult and larvae *Drosophila* (purple). Lung cancer is targeted to the trachea of larvae *Drosophila* (green) and colorectal cancer is targeted to the midgut (orange) and hindgut (blue) of adult *Drosophila*.

The homologous nature of *Drosophila* and humans allows for the study of many cancer types in *Drosophila*. The systems used to study these cancers are not based on identical structural representation but rather genetic and molecular similarities between *Drosophila* and humans (Figure 2). Selected regions of *Drosophila* are modeled to represent the target cancer type. These regions are indicative of the genes and underlying mechanisms that are homologous in humans. Cancer represented in the *Drosophila* models is also applied to distinct stages in the life-cycle stage of the fly; larvae or adult, depending on the research particularities. The current cancer types modeled in *Drosophila* include, but is not limited to, colorectal, lung, thyroid, and brain cancer, which will be addressed in this article. Furthermore, molecular elements associated with several cancer types are also studied in these *Drosophila* models including tumor suppressors, chromatin regulators, cellular growth control, tumor microenvironment, tumor invasion, and metastasis (Rudrapatna et al., 2012).

**FIGURE 3:**
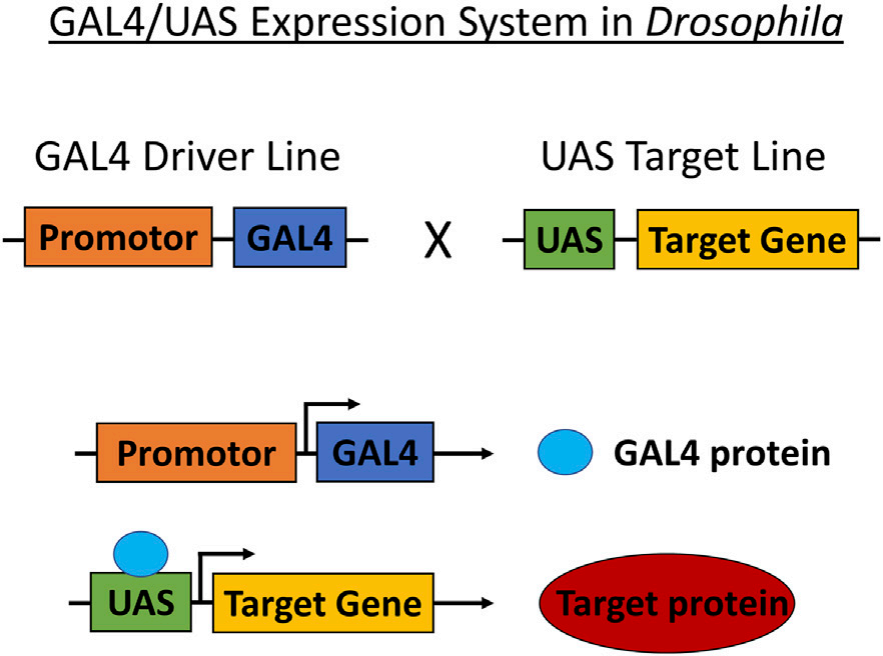
GAL4/UAS expression system in *Drosophila.* The GAL4/UAS system containing the *GAL4* transcription factor (driven by cell-or tissue-specific enhancer/promoter) and *UAS* target used for targeting genetic manipulation in *Drosophila.* Transgenes are carried by crossing parent flies transferring either *Enhancer*-*GAL4* or *UAS-target gene* that produces offspring translating the target protein.

Transgenic *Drosophila* strains are typically modeled for cancer through the use of the GAL4/UAS system. Transgenes are carried by crossing parent flies transferring either the *Enhancer*-*GAL4* or *UAS-target gene* that produces offspring with the induced target gene (Figure 3). Target gene selection is based on gene and pathway similarities corresponding to *Drosophila* and humans. The expression of the target protein generates a cascade of reactions specific to the cancer type. These reactions are observed and studied in conjunction with anticancer therapeutics (Table 1). *Drosophila* cancer models successfully identified several compounds capable of reducing cell proliferation from various Food and Drug Administration (FDA) approved chemical compounds, indicating the strong cancer-related chemical screening potential of using *Drosophila* models in anticancer research.

**TABLE 1:** *Drosophila* cancer models and drug treatment. *Drosophila* cancer models are described based on cancer type, mammalian mutation, corresponding *Drosophila* mutation and drug treatment of the *Drosophila* model.

Mammalian mutation	*Drosophila* mutation	Drug treatment in *Drosophila*	References
Colorectal cancer			
*KRAS* ^*G12V*^ *, TP53, PTEN, APC, and SMAD4*	*RAS* ^*G12V*^ *, p53* ^*RNAi*^ *, PTEN* ^*RNAi*^ *, APC* ^*RNAi*^ *, and SMAD4* ^*RNAi*^	SC79 then BEZ235	Bangi et al (2016)
		Bortezomib then BEZ235	
*Axin 1 and Axin 2*	*Axin*	Oxazole	Gonsalves et al (2011)
		Thiazole	
		Thiazolidinedione	
*KRAS* ^*G12V*^ *, APC*	*RAS* ^*G12V*^ *, APC* ^*N175K*^	Oxaliplatin	Adams et al (2021)
		5-Fluoro-5′-deoxyuridine	

Lung cancer			
*KRAS* ^*G12V*^ *, PTEN*	*RAS* ^*G12V*^ *, PTEN* ^*RNAi*^	Trametinib and fluvastatin	Levine and Cagan (2016)
*KIF5B-RET*	*KIF5B-RET*	Sorafenib and erlotinib	Das and Cagan (2017)
		Sorafenib and paclitaxel	
*EGFR* ^*CA*^	*EGFR* ^*CA*^	Afatinib	Bossen et al (2019)
		Gefitinib	
		Ibrutinib	
		Bazedoxifene and afatinib	
*KRAS*	*RAS, RAS85D* ^*e1B*^	Gefitinib and erlotinib	Aritakula and Ramasamy (2008)

Thyroid cancer			
*RET* ^*M198T*^	*dRET* ^*C695R*^ *, dRET* ^*M1007T*^	ZD6474	Vidal et al (2005)
		AD57	
		AD58	
		AD80	
		AD81	
*RET*	*dRet* ^*MEN2B*^	Sorafenib	Dar et al (2012)
		LS1-15	
		APS3-69-1	
		APS5-16-2	
		APS6-45	

Brain cancer			
*LgL1 and LgL2*	*[l(2)gl]*	Artemisinin curcumin	Das et al (2014)

### 2.1 Colorectal cancer

Colorectal cancer (CRC) has the second-highest cancer-related mortality rate, with 1.9 million new cases and 935,000 deaths reported globally in 2020 (Sung et al., 2021). The development of CRC is caused by chromosomal abnormalities, gene mutations, and epigenetic alterations of genes regulating apoptosis, angiogenesis, proliferation, and differentiation (Vogelstein et al., 1988). In particular, the activation of oncogenes (*KRAS* and *BRAF*) and the inactivity of tumor suppressors (*APC*, *p53*, *DCC,* and *p16*), as well as mismatches during gene repair (*MLH1* and *MSH2*), contribute to the development of the disease (Tanaka et al., 2006). The *RAS* gene family is especially well studied due to reports that 30–50% of colorectal tumors contain mutant *KRAS* oncogenes, while mutations in oncogenic *RAS* isoforms, such as *NRAS* and *HRAS*, are responsible for an estimated additional 6% of CRC tumors (Valtorta et al., 2013; Chang et al., 2016). Current therapeutic approaches targeting RAS-related tumors show limited efficacy, particularly in patients with KRAS-mutant metastatic CRC (Nazarian et al., 2010; Misale et al., 2012; Stephen et al., 2014). These poor therapeutic results are due to the rising resistance of late-stage CRC tumors to targeted therapies.

Safeguarded genes and pathways associated with CRC have been analyzed extensively in cell cultures and mouse modules (Sancho et al., 2004; Markowitz and Bertagnolli, 2009). The *Drosophila* hindgut distinctly retained these CRC-related pathways, presenting a near-identical composition of the cell populous to the mammalian colon (Takashima et al., 2008; Fox and Spradling, 2009). Similarly, the *Drosophila* midgut represents functions and molecular characteristics exhibited in the human intestine (Sadaqat et al., 2021). The intestinal maintenance of both *Drosophila* and mammals is controlled by proliferating cells that produce post-mitotic secretory cells and absorptive enterocytes (Casali and Batlle, 2009). These genetic similarities between *Drosophila* and mammals allow for the successful modeling of CRC in *Drosophila*. The *Drosophila* models demonstrate key hallmarks of human CRC, such as disruption of cellular differentiation, amplified cell proliferation, and decreased intestinal homeostasis (Gonzalez, 2013; Martorell et al., 2014; Villegas, 2019). The models also permit a whole-organism assessment of various CRC anticancer therapies.

Data analyzed from the Cancer Genome Atlas (TCGA) shows that 90% of CRC patient samples present mutations in two or more signaling pathways. Recurrent mutations specifically affect *TGF-β*, *WNT*, *PI3K*, *RAS/MAPK*, and *p53* pathways (Bangi et al., 2016). *Drosophila* was effectively used by Bangi and colleagues (2016) to model 4 or 5 recurrent mutations based on CRC genotypes. The mutations reflected patient-specific genes identified through TCGA analysis corresponding to the 212 human colon tumors. The GAL4/UAS expression system allowed for the selected genes to be altered via transgene expression and tissue-specific RNAi (Figure 3). The authors combined active *RAS* ^*G12V*^ with RNA interference (RNAi) knockdown of tumor suppressors *p53*, *APC, PTEN,* and *SMAD4* (Bangi et al., 2016)*.* Transgenes were targeted to the adult *Drosophila* hindgut epithelium. Principle colon cancer pathologies were cataloged, including cellular proliferation, basement membrane disruption, apoptosis and senescence circumvention, distant metastasis, and epithelial-mesenchymal transition (EMT). Specifically, *RAS* ^*G12V*^, *p53* ^*RNAi*^, *PTEN* ^*RNAi*^ *,* and *APC* ^*RNAi*^ mutations produced more peracute phenotypes.

Furthermore, the study by Bangi and colleagues (2016) accentuates the potential use of *Drosophila* in personalized medicine. These selectively mutated flies were used to test the effect of FDA-approved cancer drugs in CRC. The *Drosophila* models showed responses to oncogenic reagents based on grouped patient genotypes. The authors reported inhibition of the tumor suppressor mTORC1 with the activation of the *RAS* oncogene and simultaneous loss of the tumor suppressor *PTEN* (Bangi et al., 2016). This indicates possible CRC resistance to PI3K/mTOR inhibitors. Particular interest was set on BEZ235, the first PI3K/mTOR inhibitor drug to enter into clinical trials (Peyton et al., 2011). Mimicking clinic trial results, BEZ235 failed to diminish the spread of CRC in the *Drosophila* models. The efficacy of BEZ235 was, however, considerably enhanced with the addition of another FDA-approved cancer drug. Pretreatment of the CRC *Drosophila* models with SC79 (AKT-activating compound) followed by BEZ235 successfully reduced the spread of CRC. This two-step therapy also proved effective with the pretreatment of bortezomib (a proteasome inhibitor) followed by BEZ235. Interestingly reversing the drug order rendered the treatment ineffective, demonstrating the importance of the action mechanisms of drugs. Many genetically manipulated *Drosophila* model combinations were tested using sixteen anticancer drug compounds specific to *RAS* mutations (Bangi et al., 2016). Synergistically acting compounds demonstrated restored sensitivity against PI3K pathway inhibitors and an increase in mTORC1 activity. This was observed in *Drosophila* CRC models, mammalian CRC cells, and a CRC GEMM (genetically engineered mouse model) (Bangi et al., 2016).

Simpler gene families in *Drosophila* reduce potential complexities in loss-of-function studies, as fewer genes are modulated. Specifically, *Drosophila* offers a unique experimental state as it has only one Axin, as appose to the Axin 1 and Axin 2 present in mammalian cells. In a study by Gonsalves and colleagues (2011), the authors focused on the *Drosophila* loss-of-function mutation on this Wg/Wnt pathway inhibitor, namely Axin. The *Drosophila Wg* (*wingless*) gene, is a homolog of the mammalian *WNT* (*wingless-related integration site*) gene. These are structurally related genes that encode proteins implicated in developmental processes, such as patterning during embryogenesis and cell fate regulation, as well as oncogenesis (Miller, 2001; Clevers, 2006). In the absence of a Wg/Wnt ligand, Axin (forming part of a protein complex) controls β-catenin degradation. The Wg/Wnt ligand segregates Axin, causing β-catenin stabilization and, subsequently, Wg/Wnt transcription activation. The authors observed continuous Wg signal activation by depleting *Drosophila* cells of Axin, thereby detecting β-catenin stabilization. Stabilization of β-catenin is essential to CRC-linked tumorigenesis (Morin et al., 1997; Johnson et al., 2005). Mutations related to the regulation of this β-catenin transcriptional activator led to the accumulation of β-catenin in the nucleus, where the molecule causes uncontrolled gene transcription. The build-up of β-catenin in the nucleus is reported in 80% of CRC tumors (White et al., 2012).

Gonsalves and colleagues (2011) screened a small-molecule library to identify three small-molecule inhibitors of the Wg/Wnt pathway, particularly β-catenin responsive transcription inhibitors. The inhibitors (oxazole, thiazole, and thiazolidinedione) modeled in the *Drosophila* cells were shown to stabilize β-catenin by inhibiting β-catenin/TCF complex formation. These three drugs were tested in the transgenic *Drosophila* cells, human colon cancer patient biopsies, and human colon cancer cells (HCT-116 and HT29). The inhibitors proved cytotoxic in human colon cancer cells, non-toxic to the *Drosophila* cells, and when tested in human colon cancer patient biopsies, they indicated an efficacy comparable to FDA-approved anticancer drugs (Gonsalves et al., 2011). The study by Gonsalves and colleagues (2011) imitates the constitutive Wnt activity presented in cancers absent of tumor suppressor adenomatous polyposis coli (APC) (Rowan et al., 2000; Fearnhead et al., 2001). Similar to Axin, APC also acts to inhibit Wg/Wnt pathways. However, APC is a negative regulator of Wnt that controls Wnt signaling by blocking β-catenin in the nucleus. The oncogenic applications of APC inhibitor-related research are paramount, with 80–85% of sporadic colorectal tumors being exclusively associated with APC mutations (Zhang and Shay, 2017).

Accurate modeling of CRC in *Drosophila* models was also shown by clonal activation of *RAS* and *Wg/WNT* pathways in the adult *Drosophila* midgut (Wang et al., 2013; Martorell et al., 2014). Similarly, other studies have shown that *Drosophila* CRC models with these *RAS* and *Wg/WNT* mutations effectively identify cancer-triggering mutations (Gonzalez, 2013; Villegas, 2019). Combined *RAS* ^*V12*^ and *WNT* signaling pathway mutations are known as habitual CRC initiators in the *Drosophila* midgut (Jackstadt and Sansom, 2016). Mutations of the APC disrupt the Wg/Wnt pathways in the intestinal epithelia, while overexpression of the *RAS* oncogene causes tumor-like overgrowths (Martorell et al., 2014). The analysis of these genetic mutations typically includes dissection followed by the manual joining of midgut tumor images. The clonal area is then quantified with the number of clones and other required parameters by software-dependent image analysis (Martorell et al., 2014; Suijkerbuijk et al., 2016; Ngo et al., 2020). This method is effective; however, it is also tedious and time-consuming, limiting large-scale experimentation and rapid results.

Alternatively, Adams and colleagues (2021) developed an innovative, fast, and simple method for the quantification of tumor formations. The *Drosophila* CRC models used by the authors contained *APC* ^N175K^ *-RAS* ^*G12V*^ mutated tumor cells that were marked with two reporter genes: *GFP* and *luciferase*. The clones were generated using the GAL4/UAS expression system. The introduction of a second *UAS-luciferase* transgene reporter, in addition to *UAS-GFP*, allowed for two distinct screening methods. The first method relies on the sensitive luciferase-based assay used to test tumor formation. The luciferase activity is reported using a microplate reader, providing rapid batch processing of whole organisms. The second method uses the GFP reporter for clone visualization and quantification. Images are created with custom-designed macros used in combination with Fiji, an extensively available imaging software. This method provides an automated analysis of the total *Drosophila* midgut presented with clones. Particular attention was set on clone size, which was determined by the authors as the most pragmatic method for identifying the probability of clones conforming to tumors.

Following genetic alterations, the *Drosophila* CRC models were screened and tested with CRC-related drugs; oxaliplatin and 5-fluoro-5′-deoxyuridine. The current systems employed in identifying new CRC therapies are traditionally small molecule screening techniques based on computational software or cell culture followed by enzymatic assays. These systems present favorably during *in vitro* testing; however, they proved ineffective for toxicity screening in the proceeding whole-organism mammalian model experiments (Horvath et al., 2016). The *Drosophila* CRC models designed by Adam and colleagues (2021) also assisted in the development of a rapid drug-and genetic screening system. Quantified data collected from the *Drosophila* CRC models were used successfully to validate the model’s rapid response to the two standard CRC drug treatments. The introduction of a second *UAS-luciferase* transgene reporter, in addition to *UAS-GFP*, enabled the high-throughput screening tool in *Drosophila*.

### 2.2 Lung cancer

Statistically, lung cancer has the highest mortality rate among all cancer types, with 1.8 million deaths and an additional 2.2 million new cases reported in 2020 (Sung et al., 2021). Approximately 85% of lung cancer cases present with non-small-cell lung carcinoma (NSCLC) (Griffin and Ramirez, 2017). Conventional lung cancer treatments generally include chemoradiotherapy with complimenting targeted therapies. However, the current drugs targeting lung cancer have proven ineffective in tumor suppression while inducing toxicity and drug resistance. The homologous molecular factors and epithelial cellular similarities between the *Drosophila* tracheal and vertebrate lung development are significant (Andrew and Ewald, 2010; Behr, 2010; Roeder et al., 2012). The multi-branched tubular structure of the *Drosophila* tracheal system consists of larger primary tubes that branch out into smaller diameter branches and end in terminal sectors. This forms an interconnected hierarchy of tubes, analogous to vertebrate lungs, with both providing oxygen to the organism. The development of the *Drosophila* tracheal branching system is distinctly dependent on Fibroblast Growth Factor (FGF) signaling (Ghabrial et al., 2003; Grifoni et al., 2015). Tracheal branching in *Drosophila* is distinctly triggered by the FGF receptor homolog *breathless* (*btl*). The FGF signaling also initiates the development of the vertebrate lung branching system (Bellusci et al., 1997; Park et al., 1998).

*Drosophila* was used by Levine and Cagan (2016) to model lung cancer with mutated *RAS* ^*G12V*^ and *PTEN* (PI3K negative regulator) knockdown. The *Drosophila* model targeted the *btl* gene using the GAL4/UAS expression system (Figure 3). Genetic alterations to the trachea caused tumor-like growth, tracheal cell proliferation, and early larval stage model death. Mutation selection was based on the association of active oncogenic *RAS* isoforms, commonly affiliated with active PI3K pathway signals (Cancer Genome Atlas Research Network, 2012; Kandoth et al., 2013). Chemical screening of 1192 FDA-approved drugs (chosen to reduce model lethality) identified trametinib, the MEK1/2 kinases inhibitor drug, and fluvastatin, the HMG-CoA reductase inhibitor drug used in cardiovascular treatment. Synergistically, these two compounds respectively inhibited RAS and PI3K pathway activity. This led to decreased over-proliferation, reduced whole-organism toxicity, and inhibited the growth of A549 human NSCLC cells with activated *RAS* ^*G12V*^ (Levine and Cagan, 2016). Trametinib is currently the only MEK inhibitor with FDA approval for NSCLC treatment as a monotherapy or in combination with dabrafenib. Clinical treatment of trametinib and dabrafenib in NSCLC patients indicates survival benefits and continued response to therapy (Planchard et al., 2022). While fluvastatin has been indicated in various cancer research, the drug is not FDA approved for anticancer treatment. Consequently, the synergistic treatment of trametinib and fluvastatin in NSCLC patients is unknown.

Additionally, *Drosophila* facilitated the creation of novel therapeutic strategies focused on the multiple gene fusion oncogene *KIF5B-RET* (Das and Cagan, 2017). Identified as a critical lung cancer driver, the structure of this KIF5B-RET oncoprotein suggests that simultaneous multi-kinase activation is therapeutically required (Ju et al., 2012; Kohno et al., 2012; Lipson et al., 2012; Takeuchi et al., 2012). Reported primarily in non-smoker patients with few genetic alterations in notable cancer agents, *KIF5B-RET* account for an estimated 2% of NSCLC cases (Takeuchi et al., 2012). Previous research done by Levinson and Cagan (2016) optimized *CCDC6-RET* and *NCOA4-RET* genes to generate transgenic *Drosophila* models. They used the GAL4/UAS expression system in a thyroid-linked cancer study, concluding with an unknown pathway of RET fusion activation (Levinson and Cagan, 2016). Accordingly, Das and Cagan (2017) focused on activation pathways associated with the RET fusions to generate a patient-derived *KIF5B-RET* mutation. This selected mutation was also expressed in *Drosophila* epithelium through the binary GAL4/UAS system. The study identified canonical signaling pathway activation through the C-terminal RET kinase domain of KIF5B-RET. The N-terminal domain of KIF5B also activated multiple receptor tyrosine kinases (RTKs), including fibroblast growth factor receptor (FGFR) and epidermal growth factor receptor (EGFR) signaling (Das and Cagan, 2017). These findings offer valuable insight into pathway vulnerabilities requiring multi-targeted agents of selected therapeutic cocktails. Administration of drugs designed singularly for RET inhibition proved ineffective in *KIF5B-RET* transformed cells. However, in combining the RET inhibitor sorafenib, with the EGFR inhibitor erlotinib or microtubule inhibitor paclitaxel, high efficacy was demonstrated in human cell line *KIF5B-RET* models and *Drosophila KIF5B-RET* models alike (Das and Cagan, 2017). While these *KIF5B-RET*-positive NSCLC therapies await patient study validation, therapeutics of other fusion kinases may advance through *Drosophila* signal pathway activation investigations.

Effective NSCLC treatment is frequently associated with simultaneously activated pathway inhibitors. However, the activation and preservation of NSCLC are limited to relatively few oncogenic driver mutations, with EGFR as the most notable lung cancer oncogene (Kandoth et al., 2013; Rotow and Bivona, 2017). Oncogenic *KRAS* mutations are established prime promoters of NSCLC and are known to incite resistance to secondary cancer therapies and EGFR inhibitors (Riely et al., 2009). The *KRAS* proto-oncogene functions in cascade signal transductions initiated by EGFR binding (Graziani et al., 1993). EGFR participates in a complex of signaling pathways and developmental processes. Structurally, EGFR contains an intracellular tyrosine kinase (TK), with the *Drosophila* TK domain presenting distinct similarities to humans (Bier, 2005). Additionally, EGFR signaling in *Drosophila* is vital to the organism’s eye structure, throughout developmental stages, and to the wing development, during the late third instar larval stage (Guichard et al., 1999). Overexpression of EGFR accounts for up to 80% of NSCLC cases (Molina et al., 2008).

A *Drosophila* lung tumor model was designed to target ectopic expression of constitutively active *EGFR* isoform (*EGFRCA*) in the airway epithelium of *Drosophila* (Bossen et al., 2019)*.* Extensive phenotyping of the NSCLC model induced massive hyper- and metaplasia. Modified *blt-GAL4>UAS-EGFR* ^*CA*^ expression promoted death in later larval development stages. Furthermore, *ppk4-GAL4>UAS-EGFR* ^*CA*^ expression prompts early third instar stage death. Model death caused by alterations to the *pickpocket* (*ppk*) gene is presumably from oxygen deficiency. The authors quantified these *Drosophila* larval mortality readouts to screen numerous possible combinations of NSCLC therapies from 1000 FDA-approved compounds (Bossen et al., 2019). Drug screening identified TK inhibitor (TKI) compounds afatinib, gefitinib, and ibrutinib. These compounds proved capable of rescuing lethality in the *Drosophila* whole-organism model. They also reversed structural trachea phenotypes with afatinib and gefitinib, showing impressive survival rates (Bossen et al., 2019). Furthermore, secondary screening of the pharmacologic FDA-approved library was performed. Bazedoxifene and afatinib were identified during this drug screening as potential synergistically acting compounds. Combined, these two compounds were able to rescue EGFR-induced lethality by reducing hypoxia-inducing JAK/STAT signals.

Another study used *Drosophila* lung cancer models as an *in vivo* drug screening system to analyze EGFR-associated TKI pathways via enhancer-suppressor assays (Aritakula and Ramasamy, 2008). Mutations and transgenics relating to EGFR pathways are well documented, providing an optimal system for drug target identification, target validation, and secondary effect determination research. The lung cancer *Drosophila* model was developed using the GAL4/UAS system. The gain-of-function analysis included upstream activation sequences: *UAS–EGFR* ^*λtop*^, *UAS-Argos,* and *UAS-CycE*. Simultaneously, tissue-specific GAL4 gene drivers included *vg*-GAL4, *ey*-GAL4, and *omb*-GAL5 with a lethal recessive allele of *RAS* and *RAS85D* ^*e1B*^ used for loss-of-function RAS-related pathways (Aritakula and Ramasamy, 2008). Model evaluations focused on mechanisms by which gefitinib and erlotinib (TKIs) block *Drosophila* EGFR signaling. Enhancer-suppressor analysis and *in silico* analysis were used for evaluations. The selection of the FDA-approved gefitinib and erlotinib TKIs was based on the reported efficacy of these drugs in chemotherapeutic treatments of cancer patients with EGFR-induced tumors (Maemondo et al., 2010; Ciuleanu et al., 2012). Gefitinib and erlotinib combined showed suppression of EGFR-induced eye phenotypes in *Drosophila*. On its own, gefitinib suppressed EGFR-induced wing phenotypes. Both drugs act to inhibit diphosphorylated forms of the extracellular signal-regulated kinase (dp-ERK1/2) in both the eye and wing imaginal discs of wild-type larvae models (Aritakula and Ramasamy, 2008).

### 2.3 Thyroid cancer

In 2020, thyroid cancer was responsible for an estimated 44,000 deaths worldwide and 586,000 new cases (Sung et al., 2021). Globally, a rise in thyroid cancer cases is reported yearly, particularly from developed countries (Kim et al., 2020). Thyroid cancer presents a wide range of complexities and mutations (Vidal et al., 2005; Das and Cagan, 2017). Mutations in the RET receptor tyrosine kinase (RTC), a 120 kDa transmembrane protein, are responsible for the development of thyroid cancer in *Drosophila* and humans. Additionally, mutations that trigger RET activity led to *multiple endocrine neoplasia type 2A* and *2B* (*MEN2A* and *MEN2B*) and familial medullary thyroid carcinoma (FMTC) thyroid cancers (Vidal et al., 2005).

Vidal and colleagues (2005) developed transgenic *Drosophila* models expressing mutated *RET (dRET)* isoforms using the GAL4/UAS system (Figure 3). This thyroid cancer model was created to mimic *FMTC*, *MEN2A*, and *MEN2B*- like isoforms. The mutated *dRET* isoforms were targeted to the developing eye in adult *Drosophila*, as flies do not have a thyroid (Figure 2). The authors successfully generated the *Drosophila* genotypes (*GMR-dRet* ^*M559T*^ and *ptc-dRet* ^*M955T*^) to mimic thyroid cancer in *Drosophila* (Vidal et al., 2005). The selection of the *RET* ^*M198T*^ mutation was based on known medullary thyroid carcinoma (MTC) patient genetics. The *MEN2A*-associated mutation was mimicked by replacing cysteine with arginine at position 695 (C695R). The *MEN2B*-associated mutation was mimicked by engineering a methionine-to-threonine point mutation into a full-length *dRET* cDNA at codon 1,007. Altered *dRET* isoforms were each cloned behind the eye-specific glass multiple reporter (GMR) promoter. The stable transgenic fly lines were generated by standard methods to create *GMRd-RET, GMR-dRET* ^*C695R*^ *,* and *GMR-dRET* ^*M1007T*^ *Drosophila* thyroid cancer models. These newly generated models expressed wild-type, *C695R,* or *M1007T dRET* isoforms, respectively, in their developing eyes.

Vandetanib, also known as ZD6474, was originally identified as a chemical inhibitor of the vascular endothelial growth factor receptor 2 (VEGFR2) RTK, with additional activity against EGFR (Vidal et al., 2005). ZD6474 is an anticancer medication used for the treatment of thyroid gland tumors. The drug acts as a kinase inhibitor of cell receptors such as the VEGFR, EGFR, and the RET-tyrosine kinase. *Drosophila* thyroid cancer models were given different concentrations of ZD6474. An increase in mortality of *MR-dRET* was observed in higher concentrations of ZD6474 administration. Low concentrations of ZD6474 led to a partial rescue of *GMR-dRET, GMR-dRET* ^*C695R*^, and *GMR-dRET* ^*M1007T*^ models. Following treatment with higher doses of ZD6474, there were no phenotypically observable differences in the eyes of the thyroid cancer and wild-type *Drosophila* models (Vidal et al., 2005).

The authors could not establish the ability of the drug to rescue different isoforms, as the exact concentration of ZD6474 that entered the fly system was not obtained (Vidal et al., 2005). However, the initial phenotypes were comparable. ZD6474 is less effective in *Drosophila* isoforms of EGFRs namely, *RAS* and *RAF*. Phenotypes presented in the *Drosophila* models were not rescued by ZD6474. According to Vidal and colleagues (2005), ZD6474 either suppresses dRET activity independent of the *RAS* pathway signaling or the drug acts upstream of *dRAS1* and *dRAD*. These results support previous studies that ZD6474 acts directly on the receptor; consequently, inhibition of downstream cytoplasm kinases linked to *RAS* signaling is likely minor. The study concluded that ZD6474 acts as an *in vivo* inhibitor of the *RET* signaling pathway. *RET*-dependent phenotypes observed in *Drosophila* are strongly suppressed by ZD6474. Targeting chemical kinase inhibitors to tissues with oncogenic *RET* may offer a viable treatment for RET-dependent cancers (Vidal et al., 2005; Das and Cagan, 2017). ZD6474 completed phase three clinical trials in 2010 and became the first FDA-approved chemotherapy for *RET*-based thyroid tumors in 2011 (Wells et al., 2010). The approval of this drug validated the potential use of *Drosophila* cancer models as a powerful tool for anticancer drug discovery. However, it should be noted that ZD6474 has been reported to have high toxicity and resistance issues (Dar et al., 2012; Das and Cagan, 2017).

Furthermore, Dar and colleagues (2012) utilized the transgenic *dRet* ^*MEN2B*^ *Drosophila* model for a whole-organism efficacy validation of ZD6474 in the treatment of medullary thyroid carcinoma (MTC) patients. The authors improved the efficacy of ZD6474 for drug screening by developing a quantitative viability assay that uses the GAL4-UAS expression system. The cancer models targeted *dRet* ^*MEN2B*^ in developing *Drosophila* eye, wing, and leg. The *ptc˃dRet* ^*MEN2B*^ assay results indicated that the patched promoter is responsible for oncogene expression of developing epithelial and other tissues. The *dRet* ^*MEN2B*^ *Drosophila* model was used to structurally modify ZD6474. The modified ZD6474 generated improved anticancer targets, including AD57, AD58, AD80, and AD81 (Dar et al.,2012). The alteration showed minimal toxicity and improved efficacy (Dar et al., 2012; Das and Cagan, 2017).

Another study developed *Drosophila* MTC models to access novel targets and chemical space from the clinical kinase inhibitor sorafenib (Sonoshita and Cagan, 2017). The combination of chemical and genetic modification screening with computational modeling showed that kinases strongly enhance or limit the activity of sorafenib. Previous studies found that sorafenib inhibits RET, BRAF, and KDR/VEGFR19 (Wilhelm et al., 2004). However, sorafenib was progressively refined, and the results were a new class of kinase inhibitors (LS1-15, APS3-69-1, APS5-16-2, and APS6-45) with distinct polypharmacology and an improved therapeutic index in *Drosophila* and human MTC xenograft models.

### 2.4 Brain cancer

Cancer of the brain and nervous system accounted for approximately 308,000 new cases and 251,000 deaths in 2020 (Sung et al., 2021). Brain cancer affects young and old individuals and contributes to high mortality rates worldwide with limited therapeutic options (Sampson et al., 2020). The central nervous system (CNS) shows remarkable evolutionary conservation in cellular composition and neuro-developmental mechanisms (Bilen and Bonini, 2005). Gliomas account for almost 80% of all malignant primary CNS tumors (Hanif et al., 2017). Glioblastoma (GBM) tumors are the most common and aggressive CNS tumors. GBM tumors infiltrate the brain and proliferate rapidly with limited therapeutical therapies currently available (Read et al., 2009; Sonoshita and Cagan, 2017; Yamamura et al., 2021). Understanding the genetic and molecular rationale underpinning gliomagenesis can assist the development of effective anticancer therapeutics.

The mutation of the EGFR tyrosine kinase is the most common genetic lesion in gliomas. Glioma-associated EGFR mutant forms have constitutive kinase activity, which drives cellular proliferation and migration by persistently stimulating Ras signaling (Maher et al., 2001; Furnari et al., 2007). Loss of the lipid phosphatase PTEN, which inhibits the phosphatidylinositol-3 kinase (PI3K) signaling pathway, also causes glioma formation. Furthermore, activating mutations in PIK3CA, which encodes the p110 catalytic subunit of PI3K, are another prevalent genetic disease. Akt, a key PI3K effector, is frequently constitutively activated in gliomas (Maher et al., 2001; Furnari et al., 2007). Multiple mutations that coactivate the EGFR-Ras and PI3K-Akt pathways are required to cause glioma. EGFR-Ras or PI3K mutations alone are not sufficient to convert glial cells (Holland et al., 2000; Maher et al., 2001). Understanding how these mutations interact with the tumors’ neurodevelopmental origins could lead to new insights into the mechanisms of gliomagenesis. Multiple glial cell types maintain proliferative capacity in the mammalian brain, including differentiated astrocytes, glial progenitors, and multipotent neural stem cells. Many developmental processes in these cell types are regulated by EGFR-Ras and PTEN-PI3K signaling, including proliferation and self-renewal, which are also characteristics of glioma cells (Furnari et al., 2007). *Drosophila* has multiple cell types that require EGFR pathway signaling for normal development. Brain cancer can be studied using glia cells in the instar larvae or adult fly. These cells are homologous to mammalian glia units of development with comparable gene expression and function (Freeman and Doherty, 2006).

Read and colleagues (2009) developed a glioma *Drosophila* model by generating mutant phenotypes through hyperactivation of these pathways in *Drosophila* glia and glia precursors. EGFR (dEGFR), Raf(dRaf), PIK3CA(dp110), PTEN (dPTEN), and Akt (dAkt) each have a single functioning ortholog in *Drosophila*, and Ras(dRas85D, dRas64B) has two functional orthologs (Read et al., 2009). The authors used the GAL4/UAS expression system (Figure 3) to undertake glial overexpression tests using the repo-Gal4 driver. The repo-Gal4 driver provides continuous UAS-transgene expression in almost all glia from embryos through adulthood (Brand and Perrimon, 1993). The authors used the UAS-dsRNA constructs for glial-specific RNAi, which they confirmed with phenotypic testing and/or antibody labeling (Dietzl et al., 2007; Read et al., 2009). Constitutive co-activation of the EGFR-RAS and PI3K pathways in *Drosophila* brain cancer models stimulated glial neoplasia, which resulted in highly proliferative and invasive neoplastic cells. The co-overexpression of activated dEGFR (dEGFR^λ^) and dp110 (dp110^CAAX^) via repo-Gal4 led to a 50-fold increase in glia. Co-overexpression of dEGFR and PI3K pathway core components, such as dAkt, resulted in phenotypes comparable to repo > dEGFR^λ^;dp110^CAAX^. The phenotypes did; however, differ depending on the pathway activation level and transgene expression. Co-overexpression of constitutively active dRas (dRas85DV^12^) or its effector dRaf (dRaf^gof^) with dp110^CAAX^, dAkt, or a dPTENd^sRNA^, which partially knocked-down dPTEN, resulted in dramatic glial overgrowth. Finally, co-overexpression of dPTEN or dominant-negative dRas85D (dRas85D^N17^) inhibited glial outgrowth in repo > dEGFR^λ^;dp110^CAAX^ larvae. This demonstrates that Ras activity and excess phospho-inositols are required for neoplasia.

Furthermore, when the EGFR and PI3K pathways are activated together, they induce far more severe phenotypes than would be expected if individually active (Read et al., 2009). Excess glia appeared in early larval stages in repo > dEGFR^λ^;dp110^CAAX^ brains and accumulated during 5–7 days. The dEGFR^λ^;dp110^CAAX^ glia disrupts the normal cellular architecture of the brain consequently concluding that the dEGFR^λ^;dp110^CAAX^ glia are neoplastic. Read and colleagues (2009) demonstrated that *Drosophila* glia and glial precursors, constitutive coactivation of EGFR-Ras and PI3K signaling leads to neoplastic, invasive cells that form transplantable tumor-like growths, replicating human glioma and animal glioma models. This strong organotypic and cell-type-specific *Drosophila* cancer model showed malignant cells produced by mutations in hallmark genes and pathways known to be driving forces in analogous human cancer. These effectors interact together in a network to promote improper cellular growth and migration by coordinating cell cycle entry and progression, blocking cell cycle exit, and stimulating cell cycle entry and progression. While the pathways in this network are interrelated, they behave in a synergistic rather than additive manner.

Four pathway circuits were found to be necessary for glial neoplasia formation. The dRas and dMyc pathways caused dCyclinE and dCyclinD to drive cell cycle entry. Phosphorylation and inactivation of the retinoblastoma protein triggered E2F activators to stimulate cell cycle entry. The *pointed* (*Pnt*) pathway causes the *string* gene (*Stg*) to promote cell cycle progression through the activation of EGFR-Ras-Erk. Glial-specific RNAi knockdown of *Pnt* reduced stg expression and completely suppressed *dEGFR* ^*λ*^ *;dp110* ^*CAAX*^ neoplasia. Furthermore, pnt proteins are important for stg expression and over-proliferation in the *dEGFR* ^*λ*^ *;dp110* ^*CAAX*^ glia. The *Tor-elF4E-S6K* pathway increased protein translation mediated by dS6K and deIF4E. Particularly, the expression of cell cycle regulators and ribosomal components, through dMyc, the single *Drosophila* ortholog of the Myc and bHLH transcription factors. The authors presume that dMyc mediates signal integration between EGFR-Ras and PI3K pathways. *Drosophila* orthologs of *CyclinE*, *Cdc25*, and *Myc* were shown to be key rate-limiting genes required for glia neoplasia. Furthermore, orthologs of *Sin1*, *Rictor*, and *Sdk4* are genes required only for abnormal neoplastic glia proliferation.

*PTEN* loss occurs in 90% of primary glioblastomas, as a consequence of increased signaling of the PI3-kinase pathway. This pathway is also activated by mutations in the *EGFR*, *PIK3* ^*CA*^ *,* and *PIK3* ^*R1*^ genes. Activation of this pathway in turn activates atypical protein kinase C (PKC), which phosphorylates and inactivates the *lethal giant larvae* (*Lgl*) gene (Verhaak et al., 2010). Inactivity of the *Lgl* gene causes downstream *PTEN* loss and leads to lgl inactivation (Plant et al., 2003; Betschinger et al., 2005; Verhaak et al., 2010; Killela et al., 2014). Humans have two genes that are homologous to *Drosophila Lgl* gene*,* the *LgL1,* and *LgL2* (Klezovitch et al., 2004). Similar to *Drosophila*, the *LgL1* gene is phosphorylated and inactivated by downstream *PTEN* loss in human glioblastoma.

Das and colleagues (2014) developed *Drosophila* brain cancer models targeting the loss of *lethal(2) giant [L(2)gl]* gene. The P127 protein is encoded by *l(2)gl*, and has a human homolog *LLGL-1* (previously known as HUGL), which codes for LLGL. Low expression of LLGL is a known cause of colorectal cancer (Schimanski et al.,2005; Zimmermann et al., 2008). *Lgl1* knockout animal models reportedly develop severe brain dysplasia, in which neural progenitor cells fail to differentiate but continue to proliferate uncontrollably (Klezovitch et al., 2004). Mutation in the *lgl* gene promotes brain tumor formation in *Drosophila* at the larval stage of development and brain dysplasia in mice (Grifoni et al., 2013; Das et al., 2014). During the extended life of the larvae, the cerebral hemispheres and imaginal discs become several times larger than their typical size. Overgrowth of the brain tissue is caused by the over-proliferation of neuroblasts, leading to brain cancer (Woodhouse and Liotta, 2004; Gont et al., 2014). The tumor cells of *l(2)gl* mutants have many of the same characteristics as human cancers, such as loss of cellular form, tissue architecture, and differentiation ability (Gateff, 1978). *Drosophila*, that is homozygous for the recessive fatal gene *l(2)gl*, produces pupae considerably later than the wild type, yet most of them are unable to metamorphose and perish as third instar larvae (Gateff, 1978).

Various clinical attempts to improve brain cancer treatments including concurrent radiation, and chemotherapy treatments, as well as surgical interventions, have been unsuccessful. As a result, *in vivo* studies for screening possible harmless anticancer drugs were conducted. Das and colleagues (2014) created *Drosophila* brain cancer models by deleting the tumor suppressor gene, *Igl*, during larval development. The *Drosophila* early instar larvae were then fed with various concentrations of artemisinin and curcumin. Artemisinin, is an antimalarial medicine with possible antitumor qualities, and curcumin, is an excellent anticancer agent that has already been clinically proven to treat certain types of cancers (Grifoni et al., 2013). These cancers includes pancreatic cancer, colorectal carcinoma, and head and neck squamous cell carcinoma; however, the effectiveness of these molecules has not been studied intensively against brain cancer (LoTempio et al., 2005; Lu et al., 2011; Crespo-Ortiz and Wei, 2012). More specifically artemisinin is a sesquiterpene lactone with a 1,2,4-trioxane ring system extracted from *Artemisia annua* or annual wormwood. Artemisinin derivatives, such as dihydroartemisinin and arteether, have been used to treat certain cancers including leukemia, colon, melanoma, breast, ovarian, prostate, central nervous system (CNS), renal, pancreatic, osteosarcoma, and lung cancer cells (Crespo-Ortiz and Wei, 2012). On the other hand, curcumin is a polyphenol extracted from *Curcuma longa*. The anticancer properties of curcumin in preclinical and clinical studies are well documented (Reuter et al., 2011; Wilken et al., 2011; Aggarwal et al., 2013; Gupta et al., 2013; Kunnumakkara et al., 2017). The *Drosophila* model regarded 500 µM of artemisinin and 100 µM curcumin as optimum concentrations to induce apoptosis in brain cancer cells without being toxic to the normal cells. Additionally, effects on tumor inhibition with the combination treatment of artemisinin and curcumin showed positive results including an improvement in the median lifespan and locomotion response of the *Drosophila* models (Yadav et al., 2016).

## 3 Use of multi-therapeutic agents in cancer therapy

Tumor heterogeneity poses one of the biggest challenges in cancer therapy; it causes patients suffering from the same type of cancer or even the primary and secondary tumors in an individual to respond differently to the same treatments. Heterogeneity limits the efficacy of monotherapeutic agents to entirely eliminate all the cells in a tumor (Chen and Lahav, 2016). Using monotherapeutic agents in cancer therapy frequently results in the development of drug resistance in the cancer cells (Chen and Lahav, 2016; Palmer and Sorger, 2017). As cancer results from so many aberrant signaling pathways, targeting a singular pathway results in ineffective treatment, even if there is an initial positive response. To overcome this, multiple therapeutic agents targeting multiple pathways simultaneously can significantly increase drug efficacy whilst decreasing the therapeutic dosage, which potentially results in fewer unwanted side effects (Chen and Lahav, 2016).

Restrictive combinations (RCs) of drugs are an area of cancer therapy that has been focused on recently, although not tested in humans yet. RCs focus on strategic dosing and administration of drugs that aim to help spare normal cells and target cancer cells with a cytotoxic effect (Mokhtari et al., 2017). This regimen utilizes slight differences between cancer cells and normal cells, for example, the lack of or presence of a target (Blagosklonny, 2008). Another approach is drug re-purposing, where FDA approved pharmaceutical agents currently used for non-cancerous diseases are re-purposed in cancer therapy (Ashburn and Thor, 2004; Chong and Sullivan, 2007). This approach is efficient as the FDA-approved drugs have already passed clinical safety protocols, had pharmacokinetic profiles, and helped reduce the financial burden of novel drug development (Ashburn and Thor, 2004). *Drosophila* cancer models have been beneficial in preclinical studies into high-efficacy synergistic drug combinations (Levine and Cagan, 2016; Bossen et al., 2019).

There are, however, challenges in combinatorial therapies, for example, multidrug resistance in chemotherapy (Gottesman et al., 2002), cross drug resistance (Aird et al., 2002; Yardley, 2013), adverse drug interactions, and even the need for increased therapeutic doses as treatment regimens continue over time (Murayama and Gotoh, 2019). In addition, drug specifics, such as time of administration and dosage, are mainly only known for administrating the drug alone. Therefore, trial and error is often needed when drugs are combined, as the dynamic response of the combined drugs is often not predictable (Chen and Lahav, 2016). *Drosophila* is a great tool to be used in the process of combinational drug testing, as they are an easy and cheap alternative to other animal models such as mice. *Drosophila* cannot replace higher animal models; however, they can narrow down the drugs that should be taken into other animal models for further investigation.

For example, multiple studies have found combinational therapy options to enhance radiation therapy in *Drosophila*, that have been found to be effective in humans as well (Edwards et al., 2011; Gladstone et al., 2012; Stickel et al., 2015). More specifically, Edwards and colleagues (2011) found that a microtubule depolymerizing agent, namely, maytansinol isobutyrate increased sensitivity to radiation therapy in *Drosophila* and later in human colon cancer cells. Next Gladstone and colleagues (2012) as well as Stickel and colleagues (2015) investigated a protein synthesis inhibitor, namely bouvardin and found increased sensitivity to radiation therapy in *Drosophila* which transferred to increased sensitively in human NSCLC, head and neck cancer (HNC) and glioma. Another example of this is the use of an EGFR-induced lung cancer *Drosophila* model to identify an alternative combinational therapy for lung cancer patients (Bossen et al., 2019). Bossen and colleagues (2019), when investigating the EGFR-induced lung cancer *Drosophila* model, found that targeting EGFR along with STAT-signaling is a promising strategy for lung cancer therapy. Additionally, Adams and colleagues (2021) investigated a *Drosophila* CRC model’s response to currently approved chemotherapy drugs for colorectal cancer in humans. They found that the *Drosophila* CRC models responded well to both oxaliplatin and 5-Fluoro-5′-deoxyuridine, two commonly used chemotherapy drugs for colorectal cancer in humans. This helps support that *Drosophila* models are a viable candidate for screening drugs against human CRC, a notoriously hard cancer to treat. Therefore, *Drosophila* studies play a role in optimization of the use of multi-therapeutic agents in cancer therapy in order for it to reach its potential in effectively treating cancer in humans.

## 4 The use of *Drosophila* in personalized therapy

One of the biggest problems with cancer treatment currently is that there is only a limited number of FDA approved regimens that display variable effectiveness amongst different patients and usually cause a host of severe side effects (Ocana et al., 2011; DiMasi et al., 2013). Lastly, a big issue is the development of drug resistance against the limited approved drug regimens (Gottesman et al., 2002). Personalized medicine aims to treat each individual patient with the best possible drug, where they receive the optimal therapeutic benefit with the lowest possible side effects. One of the primary areas being focused on to achieve this is understanding the genetic contribution to the diseased state and how this contributes to the toxicity and efficiency of potential drugs. One big hurdle in personalized therapy is understanding which candidate genes are of therapeutic significance. This is complicated by the complex genetic landscape within a living organism; this is where *Drosophila* is a powerful tool for understanding the complex relationships within a whole-organism.

The advantages of *Drosophila* in drug discovery and development have already been addressed in this review; however, some particularly apply to utilizing them for personalized medicine screening as they quickly develop and are relatively cheap and easy to use. This lowers costs, which is vital in making personalized therapy affordable (Kasai and Cagan, 2010). *Drosophila’s* most valuable characteristic for personalized therapy, however, is by far the ability to easily manipulate their genes, as it has been seen that the activity of almost all *Drosophila* genes can be increased or decreased within nearly all cell types at any stage of development (Kasai and Cagan, 2010). The permission for rapid and inexpensive manipulation of multiple genes in *Drosophila*, as well as the ability to perform high-throughput whole-organism screening (Adams et al., 2000; Kasai and Cagan, 2010), makes it popular amongst the other model animals. Manipulation of gene expression and generation of specific mutations are becoming more accessible, quicker, and more affordable as the years result in the development of these technologies. Significantly it has been estimated that 75% of genes involved in disease causation are conserved between humans and *Drosophila* (Reiter et al., 2001; Pandey and Nichols, 2011). For cancer, several studies have identified novel therapeutic targets *in vivo* using the *Drosophila* model system (Vidal et al., 2005; Bangi et al., 2011; Levine and Cagan, 2016; Bossen et al., 2019).

It is important to recognize the multigenic nature of cancers. Two types of *Drosophila* models have been produced; firstly, an *in-silico Drosophila* Patient Model (DPM), which models the biomolecular regulation in cancer utilizing patient-specific gene expression data to develop personalized cancer therapeutic strategies (Gondal et al., 2021). Secondly is an *in vivo Drosophila* Patient Model (DPM) where the use of *Drosophila* in personalized cancer therapy has come with the creation of cancer avatars. These cancer avatar flies are genetically mutated to contain several mutations that mimic the human patient. The *Drosophila* are then used to screen drugs, including multi-therapeutic approaches (Bangi et al., 2019). These models may help identify compounds with higher whole-organism efficacy and decreased toxicity.

Briefly, a specific example is the treatment of KRAS-mutant metastatic colorectal cancer with nodules in the lungs, where the development of a personalized *Drosophila* model resulted in the identification of a two-drug cocktail of trametinib and zoledronate. Overall, the patient was treated with trametinib and zoledronate for approximately 11 months, exhibiting a maximum of 45% reduction in tumor burden. One side effect that developed was a severe rash; however, it was controlled using antibiotics and antihistamines, allowing for the continued use of the treatment regimen. The patient had to be taken off treatment due to the emergence of previously unobserved lesions. When the lesions were genetically analyzed, two new mutations were found, suggesting resistance formation to treatment (Bangi et al., 2019).

A second example was the case of a patient with advanced adenoid cystic carcinoma cancer type that originates in the salivary glands. A *Drosophila* cancer avatar was created containing selected mutations mimicking the patient. Robotics-based screening identified a three-drug cocktail (namely vorinostat, pindolol and tofacitinib) that was able to rescue transgene-mediated lethality in the *Drosophila* patient-specific line. A partial response was seen for 12 months, after which relapse occurred. Subsequent resistance was due to new genomic amplifications and deletions (Bangi et al., 2021). However, it must be noted that the patients in these two cases had already been on several different therapy regimens, and their cancer had already metastasized by the time personalized therapy had started. The positive response seen, even if not followed through, encourages the use of personalized therapy for future cases.

Lastly, a clinical trial ran between 2015 and 2020, which investigated personalized cancer therapy in patients with metastatic medullary thyroid or metastatic colon cancer (ClinicalTrials.gov Identifier: NCT02363647). This trial aimed to identify tumor mutations and incorporate them into personalized *Drosophila*. These personalized *Drosophila* would then be screened with FDA approved drugs to identify a single drug or any drug combinations that are the most effective and least toxic. The identified drug or drug combinations would then be tested in xenograft models before a board of experts choose the best therapeutic option for each individual. To date, 10 individuals have been recruited into the trial; however, no further details are supplied as the trial was suspended in October 2020, due to lack of funding.

One of the biggest obstacles to overcome, even in multi-therapeutic personalized therapy, is the development of drug resistance. Therefore, identifying potential drug resistance could be important in optimizing personalized treatments. Again, personalized *Drosophila* comes into play as it was found that they could potentially be used to identify biomarkers of drug resistance (Bangi et al., 2014). As the use of *Drosophila* in multi-therapeutic personalized therapy is still in its infancy additional research is needed to fully reach the potential of multi-therapeutic personalized therapy in cancer treatment. As well as an increase in fly scientists trained in using *Drosophila* in personalized medicine.

## 5 Technical considerations and limitations

*Drosophila* has emerged as an auspicious model for screening anticancer drugs. Notable characteristics of fruit flies such as 1) short life span and generation time, 2) low genetic redundancy, 3) high reproductive rates, 4) low maintenance costs and 5) genetic and functional similarities to humans make them attractive models for the discovery of anticancer therapeutics. However, some normal physiological and cancer-relevant mechanisms differ in *Drosophila* and humans due to several differences between the two organisms.

The most notable difference is the physiology and anatomy of the two organisms. Humans have a more complex physiology and anatomical organization compared to *Drosophila.* Tumors in humans are far more complex than those generated in *Drosophila.* Cancer fly models, therefore, produce a partial picture of the disease in humans (Willoughby et al., 2013; Richardson et al., 2015; Yadav et al., 2016). The consequence of such differences may produce pseudo-positive or pseudo-negative findings during drug screening. *Drosophila* particularly lacks the direct equivalent of the mammalian organs, such as the liver, pancreas, spleen, thymus, kidneys, lungs, and thyroid gland. However, cancers of the thyroid, lungs and brain can be modeled with *Drosophila* equivalent organs or using other organs such as the eye. A classic example is the modeling of thyroid cancer using the cells of the developing eye as described previously (Das and Cagan, 2017). Although not a perfect model for the study of thyroid cancer, it proved useful in understanding the mechanism of development and identification of potential anticancer therapeutics.

The current fly models do not adequately address tumor genetic heterogeneity and evolution observed in human patients. Tumor genetic heterogeneity and evolution play a key role in formation of aggressive and treatment resistant cancers (Pelham et al., 2020). Whilst there are significant strides made in replicating the genetic architecture of tumors in patients using fly “avatars” and improving treatment, an effort is required to create fly models that will address tumor evolution and heterogeneity in pursuit of improving cancer treatment outcomes.

*Drosophila* have a simple innate immune system whilst mammals possess complex adaptive and innate immune systems. Moreover, blood or lymphatic vessels are absent in *Drosophila*. For these reasons, the direct and indirect effects of drugs on the immune system and tumor neo-vasculature cannot be assessed (Gao et al., 2015; Yadav et al., 2016; Yamamura et al., 2021). In addition, the lack of an adaptive immune system further limits the testing of newer therapies such as immune checkpoint inhibitors (ICIs) which have shown greater success than small molecule/chemotherapeutic drugs.

The limitation posed by the physiological and anatomical differences between flies and humans, therefore, warrants additional preclinical testing in mammals such as mice prior to testing in humans (clinical trials). Drug screening using *Drosophila* can help verify the suitability of drug candidates in a whole-organism prior to testing in costly rodent experiments and clinical trials. However, *Drosophila* cannot replace rodent models. Mammalian models are still required to establish the pharmacokinetics and pharmacodynamics to validate the pharmacological activity of the drug candidate (Pandey and Nichols, 2011; Su, 2019). Although *Drosophila* may not replace mouse models, it is a more appropriate screening tool than *in vitro* cell culture and can narrow down the potential drugs to be screened in the more expensive mouse models.

The route of administration of a drug is important in the drug discovery process. Drug candidates are often diluted in *Drosophila* culture media for larvae (and adults), and the drug is ingested orally. Limitations of oral administration are that drug concentrations in the food may differ significantly from actual physiological concentrations. Other issues with this route of administration also include the taste of a drug: if a drug tastes bad, a fly is likely to not eat it. In addition, significant variability in dosage among flies as a result of various body sizes and amounts of drug ingested, as well as relatively low throughput, may further pose a limitation. It may be necessary to examine *in vivo* concentrations using high-performance liquid chromatography or mass spectrometry (Kuklinski et al., 2010). In adult flies, several routes of administration may be tested. These include vapor (e.g. ethanol) via injection in the abdomen or dropped directly onto the exposed nerve cord of decapitated flies (Moore et al., 1998; Torres and Horowitz, 1998). Following administration, drug candidates are often metabolized before making their way to target sites. The difference in the metabolism between humans and *Drosophila* can further limit the use of drugs that are toxic to *Drosophila* and not humans and vice versa. However, there is a strong correlation of toxicity between the two organisms for most drugs (Rand, 2010; Pandey and Nichols, 2011).

All of these factors, and more, accentuate the use of *Drosophila* as a screening platform for target discovery, primary small-molecule screening, or post-screening validation to narrow down a large pool of potential drug candidates prior to screening in costly mammalian models. Nonetheless, the use of *Drosophila* in target discovery and high-throughput screening remains a relevant whole-organism drug screening approach. The use of *Drosophila* improves the rate of discovery by reducing the time necessary to identify a small collection of potentially more effective lead compounds for final validation. Although *Drosophila* models can be informative in the discovery process, having a well-defined hypothesis and a thorough understanding of the limitations of the fly are absolutely critical for success.

## 6 Conclusion and future perspectives

Current anticancer therapies are often ineffective or result in the development of drug-resistance, especially when administered as a monotherapy. The discovery of new anticancer drugs involves complex experimental procedures, extensive assessment processes and high implementation costs. Generally, initial drug discovery relies on target identification using computational software and *in vitro* drug screening; however, these have been found to be frequently inefficient when applied to whole-organism models. The common fruit fly *Drosophila melanogaster* presents a valuable tool for screening and testing anticancer drugs in the preclinical stages of drug discovery. *Drosophila* present a high conservation of key genes, a low genetic redundancy, easy genetic manipulation and a rapid life cycle. In addition, *Drosophila* are easy to maintain and produce large amounts of offspring making them suitable for cost-effective high-throughput screening of anticancer therapies. Generated *Drosophila* cancer models, either specified per cancer type or personalized to patient genotype, can mediate high-throughput screening of FDA-approved non-cancer and anticancer drugs adapted to specified genetic requirements. In addition, *Drosophila* can provide valuable information on drug bioavailability and toxicity. In this way *Drosophila* can be used in personalized therapy to study a cancer model with multiple genetic mutations. They can also be used to screen for combinational therapies in a multi-therapeutic approach. These models are inexpensive and efficient, thus reducing the cost and time of anticancer drug discovery. However, the application of this organism in the anticancer drug discovery pipeline is still in its infancy, and greater awareness, skill development and application are key to fully utilizing the potential of *Drosophila* in drug discovery and development.
